# SARS-CoV-2 and Cytomegalovirus Co-Infections—A Case Series of Critically Ill Patients

**DOI:** 10.3390/jcm10132792

**Published:** 2021-06-25

**Authors:** Patrícia Moniz, Sérgio Brito, Pedro Póvoa

**Affiliations:** 1 Polyvalent Intensive Care Unit, São Francisco Xavier Hospital, Centro Hospitalar de Lisboa Ocidental (CHLO), 449-005 Lisbon, Portugal; patricia.moniz25@gmail.com; 2 Internal Medicine Department, São Francisco Xavier Hospital, Centro Hospitalar de Lisboa Ocidental (CHLO), 449-005 Lisbon, Portugal; sergioembrito@gmail.com; 3 Nova Medical School, Comprehensive Health Research Centre (CHRC), New University of Lisbon, 449-005 Lisbon, Portugal; 4 Center for Clinical Epidemiology and Research Unit of Clinical Epidemiology, Odense University Hospital (OUH), Odense University Hospital, 5000 Odense, Denmark

**Keywords:** SARS-CoV-2, Cytomegalovirus, co-infections, critical care, COVID-19

## Abstract

The SARS-CoV-2 pandemic has placed great strain on the most developed of health care systems, especially in the context of critical care. Although co-infections with cytomegalovirus (CMV) are frequent in the critically ill due to underlying immune suppression of multiple causes, the impact on COVID-19 patients remains unclear. Furthermore, severe COVID-19 has recently been associated with significant immune suppression, and this may in turn impact CMV reactivation, possibly contributing to clinical course. Nevertheless, multiple confounding factors in these patients will certainly challenge upcoming research. The authors present a case series of five patients admitted to the intensive care unit (ICU) in the context of respiratory failure due to severe COVID-19. All patients evolved with CMV reactivation during ICU stay.

## 1. Introduction

The current pandemic caused by SARS-CoV-2 virus infection has provoked an unprecedented health care burden worldwide with an abrupt demand for critical care provision and consequent strain on the intensive care unit (ICU) [1]. The experience and knowledge obtained during the past year have allowed the medical community to adapt and treat this emerging disease, but much uncertainty still prevails.

A history of cytomegalovirus (CMV) infection is very common among adults, the majority of which evolve with a period of latency characterized by a persistent control of viral replication [2]. Reactivation usually implies some type of weakened immunity which can be attributed to various etiologies [3]. Nevertheless, infection of immunocompetent patients in the ICU is well acknowledged, with the highest reactivation rates in septic patients. Furthermore, CMV reactivation is associated with higher ICU length of stay, longer need for invasive mechanical ventilation (IMV), increased risk of infections and mortality [4].

D’Ardes et al. were the first to report a case of CMV and SARS-CoV-2 co-infection [5]. Since then, the potentially adverse effects of CMV co-infection on COVID-19 outcome have been approached by recent publications [2]. However, case report publications of CMV co-infection remain relatively scarce [5,6,7,8,9], and the role of COVID-19 itself on CMV reactivation unclear.

The authors report a case series of five patients admitted to the ICU due to SARS-CoV-2 pneumonia who presented concomitant CMV infection/reactivation during ICU stay.

## 2. Case Presentation

### 2.1. Patient I

A 64-year-old male was admitted to the emergency department (ED) with a 7-day presentation of fever, dry cough, myalgia and chest pain. His past medical history (PMH) included stable human immunodeficiency virus 1 (HIV-1) infection (undetectable viral load; CD4+ cell count 321), diabetes mellitus (DM), hypertension (HT) and ischemic heart disease.

Initial diagnostic workup revealed a positive SARS-CoV-2 PCR assay, arterial blood gas examination (ABG) with hypoxemia (pO_2_ 71.1 mmHg with 3L/min of oxygen via nasal cannula) and chest X-ray with bilateral patchy lung infiltrates. He evolved with acute respiratory distress syndrome (ARDS) in the first 24 h and was transferred to the ICU, where he required invasive mechanical ventilation (IMV) for 22 days. Four days later, he presented respiratory distress and hemodynamic instability and was reintubated in the context of ventilator-associated pneumonia (VAP) due to Serratia. Laboratory reassessment revealed a lower CD4 count (114) and a positive CMV viral load (1012 UI/mL). The patient was extubated after 10 days of readmission and subsequently transferred to the medical ward. Hospital discharge occurred on day 67.

### 2.2. Patient II

A 61-year-old female presented to the ED with sustained fever and dyspnea within the last 24 h. She had a PMH of systemic lupus erythematosus with pulmonary, joint and renal involvement and chronic kidney disease (CKD) undergoing regular hemodialysis. Current medication included azathioprine and prednisolone (Table 1).

The patient presented hypotension responsive to fluid challenge and ABG with hypoxemia (pO_2_ 60.4 mmHg with 3L/min of oxygen via nasal cannula). The SARS-CoV-2 PCR assay was negative, and blood cultures were positive for *Enterococcus faecalis*. Antibiotics were started and the patient was transferred to the medical ward. Posteriorly, the PCR assay was repeated because of contact with a COVID-19 patient and turned out positive. She evolved with ARDS and was admitted to the ICU, where she initiated IMV. Plasma and bronchoalveolar lavage (BAL) CMV viral loads were positive (3528 UI/mL and 229 UI/mL, respectively). The patient died due to refractory circulatory shock on day 8.

### 2.3. Patient III

A 61-year-old male presented to the ED with a headache, fatigue and shortness of breath for the past 24 h. PMH included a heart transplant, DM, HT and CKD. Current medication included everolimus, mycophenolate mofetil, cyclosporine and prednisolone.

The patient had hypoxemia (ABG with pO_2_ 66.9 mmHg), the chest X-ray showed bilateral patchy pulmonary infiltrates and the SARS-CoV-2 PCR assay was positive. He was admitted to the medical ward, where he progressed with ARDS and therefore transferred to the ICU 2 days later. He underwent IMV for 37 days and the clinical course was complicated with septic shock in the context of a VAP due to *Proteus mirabillis* and *Klebsiella pneumoniae*, with the need for renal replacement therapy (RRT). Although CMV viral load upon admission had been negative, screening was repeated and viral load positive (518 UI/mL). A surgical tracheostomy was performed on day 35 due to ventilatory weaning failure. After readmission to the medical ward, he was discharged to a rehabilitation unit 87 days after admission.

### 2.4. Patient IV

A 77-year-old male was diagnosed with SARS-CoV-2 infection 10 days before presenting to the ED with dyspnea for the past 4 days. The patient’s PMH included DM and HT.

The patient presented signs of respiratory distress and hypoxemia (ABG with pO_2_ 56.6 mmHg 6L/min of oxygen via facial mask). Chest X-ray showed patchy bilateral lung infiltrates. He was transferred to the medical ward with a Venturi Mask 35%, where he evolved with worsening hypoxemia and was therefore admitted to the ICU the next day. The patient evolved with circulatory shock and severe hypoxemia, which motivated the initiation of IMV, in the context of concomitant bacterial pneumonia due to *Klebsiella pneumoniae*. Clinical deterioration ensued in the context of candidemia. Bronchoalveolar lavage CMV viral load positive (170 UI/mL). On day 40 of admission, the patient evolved with refractory circulatory shock and died.

### 2.5. Patient V

A 78-year-old male presented to ED with anorexia, dry cough and shortness of breath for the past 2 days. He had contact with a COVID-19 patient 6 days before. The patient’s PMH included HIV-1 infection (undetectable viral load and CD4+ cell count 743), DM with retinopathy and stage 3b CKD and HT.

The patient had hypoxemia (pO_2_ 50 mmHg with 4L/min of oxygen via nasal cannula), and a thoracic CT scan showed extensive bilateral pulmonary infiltrates. SARS-CoV-2 PCR screening was positive. He presented progressive clinical deterioration while in the ED and was transferred to the ICU. He required IMV for 48 days, and the clinical course was complicated with multiple respiratory infections and the need for RRT. CMV viral load upon admission was undetectable, but CMV viral load of bronchoalveolar lavage was positive on day 44 (108 copies/mL). A surgical tracheostomy was performed on day 24 due to a ventilatory weaning failure. He was subsequently transferred to the medical ward and discharged to a rehabilitation unit on day 83.

## 3. Discussion

We present five case reports of CMV reactivation in COVID-19 patients admitted to the ICU due to respiratory failure requiring IMV (Table 1). All patients were tested for CMV reactivation because of their respective underlying clinical severity and previous medical history of immune deficiency. The diagnosis was confirmed with CMV PCR testing (plasma or BAL), since antigen testing can be inaccurate in leukopenic patients [4]. Some patients had an initially negative CMV viral load, and therefore CMV reactivation occurred during ICU stay. All patients with the exception of patient IV, due to rapid clinical deterioration and death, began treatment with ganciclovir in the ICU. Although viral load was frequently low, given the positive PCR assay and underlying risk factors, curative therapy was initiated whenever possible. Patient III completed full-dose treatment for 3 weeks (5mg/kg IV q 12h), while all other patients began ganciclovir adjusted to renal function and RRT regimens.

It has been well acknowledged that critical illness itself can promote immune suppression, even in the absence of known immune deficiency states. This is due to an underlying complex immune system activation, composed of both pro- and anti-inflammatory responses. Recovery depends on the attainment of immunologic homeostasis, the lack of which can result in a type of secondary immune deficiency, compromising both innate and adaptive functions with a consequently increased risk of nosocomial infection [10,11]. Although CMV reactivation is quite common among the critically ill, debate still exists on whether such infection adversely affects the patient outcome or is merely an uneventful finding [4].

Recent findings associate severe COVID-19 with significant depletion of adaptive immune cells and increases in T-cell killing and immunosuppression. Critical care patients have demonstrated sustained T, NK and B cell lymphopenia and downregulation of HLA-DR expression, while increases in PD-1 have all been demonstrated in the first 7 days of ICU admission. These findings are not only worrisome but should reinforce careful evaluation of current therapeutic indications that can further hinder an effective immune response to SARS-CoV-2, such as glucocorticoids [12].

The role and the rate of CMV reactivation in SARS-CoV-2 patients are unclear. Clinical profiles that have been associated with worse outcomes in COVID-19 also prevail among patients with a higher risk of CMV reactivation. Characteristics such as older age, history of DM and cardiovascular diseases constitute such examples [2]. All five cases had ages older than 60 years, DM was highly prevalent and chronic immunosuppression either due to chronic illness or medication was also frequent.

Both HIV-infected patients presented recent negative viral loads previous to ICU admission, and significant HIV reactivation was excluded during ICU stay. Patient I had a very low viral load at the end of the ICU stay (35 copies), while patient V maintained a negative viral load.

CMV testing also occurred in patients without the aforementioned risk factors. However, these constituted a minority of ICU admissions, and CMV reactivation was not found in patients without comorbidities associated with immunosuppression. Consequently, this constitutes a limitation of our research, and the significance of SARS-CoV-2 infection in CMV reactivation cannot be clearly established based on our findings. The authors emphasize, however, that CMV reactivation can be overlooked, as can other nosocomial infections such as aspergillosis, characterized by unspecific clinical presentations. Considering the immunosuppression possibly associated with COVID-19, efforts should be made to prevent the underestimation of such infections in these patients.

## 4. Conclusions

The role of SARS-CoV-2 infection on CMV reactivation remains to be unraveled. Multiple confounding factors usually associated with immunosuppression, such as the clinical profile of older patients with multiple comorbidities, the secondary immune suppression of critical illness itself, underlying immunosuppressive treatments under investigation and probable immune suppression due to severe COVID-19 illness will certainly challenge further research.

## Figures and Tables

**Table 1:** Clinical characteristics of patients admitted to the ICU with SARS-CoV-2 infection due to respiratory failure who evolved with CMV infection/reactivation.

	Patient I	Patient II	Patient III	Patient IV	Patient V
Immunosuppression ^a^	HIV ^b^, DM ^c^	IS ^d^ therapy	IS therapy, DM	DM	HIV, DM
Time (days) (symptoms to ICU admission)	8	8	5	11	1
IMV ^e^ duration (days)	32	8	37	33	48
pO_2_/FiO_2_ (minimum)	169	105	88	105	113
First CMV ^f^ PCR ^g^ (date/result/sample)	D7 Positive Plasma	D2/D3 Positive Plasma/BAL ^h^	D3 Negative Plasma	D37 Positive BAL	D2 ** Negative Plasma
Posterior CMV PCR (date/result/sample)	-	-	D16 Positive Plasma	-	D45 ** Positive BAL
Outcome (90 day)	Home discharge	Death	Rehabilitation Unit	Death	Rehabilitation Unit

^a^ Immunosuppression besides critical illness; ^b^ HIV—human immunodeficiency virus infection; ^c^ DM—diabetes mellitus; ^d^ IS—immunosuppressive; ^e^ IMV—invasive mechanical ventilation; ^f^ CMV—cytomegalovirus; ^g^ PCR—polymerase chain reaction; ^h^ BAL—bronchoalveolar lavage; ** various plasma PCR assays remained negative throughout ICU stay. The only positive sample was obtained in BAL.

## Data Availability

No new data were created or analyzed in this study. Data sharing is not applicable to this article.
